# Supplementary material for: A C2-Domain Abscisic Acid-Related Gene, IbCAR1, Positively Enhances Salt Tolerance in Sweet Potato (Ipomoea batatas (L.) Lam.)
Source: Int J Mol Sci. 2022 Aug 26;23(17):9680. doi: 10.3390/ijms23179680 (PMC9456122; doi:10.3390/ijms23179680)
Supplement: Supplementary file 1 [file ijms-23-09680-s001.zip › ijms-1847894-supplementary.pdf]

Supplementary Table S1. Analysis of the cis-acting elements within the *IbCAR1* promoter

Site Name	Position	Sequence	Function
ABRE	＋239	ACGTG	cis-acting element involved in the abscisic acid responsiveness
ARE	－242	AAACCA	cis-acting regulatory element essential for the anaerobic induction
Box 4	＋167	ATTAAT	part of a conserved DNA module involved in light responsiveness
CAAT-box	－68	CCAAT	common cis-acting element in promoter and enhancer regions
CGTCA-motif	－88	CGTCA	cis-acting regulatory element involved in the MeJA-responsiveness
G-Box	＋337	CACGTT	cis-acting regulatory element involved in light responsiveness
G-Box	－237	CCACGTAA	cis-acting regulatory element involved in light responsiveness
GA-motif	－1221	ATAGATAA	part of a light responsive element
GATA-motif	＋1202	AAGATAAGATT	part of a light responsive element
GT1-motif	＋757	GGTTAA	light responsive element
P-box	＋862	CCTTTTG	gibberellin-responsive element
TATA-box	＋135	TATA	core promoter element around -30 of transcription start
TCA-element	－830	CCATCTTTTT	cis-acting element involved in salicylic acid responsiveness
TGACG-motif	＋88	TGACG	cis-acting regulatory element involved in the MeJA-responsiveness

Supplementary Table S2. Primers used in this study

Number	Primer name	Primer sequence (5′-3′)
1	AtAAO-F	GTGCAACAAGATCAGCCATTAG
2	AtAAO-R	GAGACTTCACCACAGGCATAG
3	AtABA2-F	GTGAGTTCGAGATGACCTTTGA
4	AtABA2-R	CAACACCTCCCACACTACATAA
5	AtZEP-F	TACAGAGGCCCGATTCAAATAC
6	AtZEP-R	TGATACACCCAGCTTCCATAAC
7	AtCAT-F	TGGGTGCATGGTTGTACTTTA
8	AtCAT-R	GTAGGGTAAGTGGGTTCACATC
9	AtLEA-F	TGACTGATGAGCACGGTAAC
10	AtLEA-R	CTGCAATGTCGCTGGTAGTA
11	AtSOD1-F	CATGCTGGTGATCTAGGAAACA
12	AtSOD1-R	CAACAGCCCTACCAACAATAGA
13	Atactin-F	TATCGCTGACCGTATGAGCAAAG
14	Atactin-R	TGGACCTGCCTCATCATACTC G
15	Hyg-F	TACTCCACCATCTCGTCCTTATT
16	Hyg-R	CTCCAGCGGTTGTAGAAGAA
17	M13-F	GTAAAACGACGGCCAGT
18	M13-R	CAGGAAACAGCTATGAC
19	α-tublin-F	CAACTACCAGCCACCAACTGT
20	α-tublin-R	CAAGATCCTCACGAGCTTCAC
21	q*IbCAR1*-F	CACCAGCGATCCTTACGTTATT
22	q*IbCAR1*-R	GGGAGATTTGGGTCTGAGATTG
23	*IbCAR1*-RNAi-1F	CATGCCATGGATGAAGAACAGAGCTTCTGTTTCA
24	*IbCAR1*-RNAi-1R	CATGATTTAAATGTATCCTTGTCATAGACTTGC

25	*IbCAR1*-RNAi-2F	GGATCCGTATCCTTGTCATAGACTTGC
26	*IbCAR1*-RNAi-2R	CCCGGGATGAAGAACAGAGCTTCTGTTTCA
27	pFGC5941-M1F	ATGACGCACAATCCCACT
28	pFGC5941-M1R	GTCCTCCCCTCTTTCTACC
29	pFGC5941-M2F	TTACTTACACTTGCCTTGGAG
30	pFGC5941-M2R	CATTAGAATGAACCGAAACC
31	IbAPX-F	GTCTTCCTGATGCCACACAA
32	IbAPX-R	ATGAGTGGGTTGGTAGTCCA
33	IbPOD-F	CATTAAGACCGCGGTTGAGA
34	IbPOD-R	GTTAGCTCCTCCCTGGTTTG
35	IbLEA-F	CCAGCATGGAAACCCCGTAT
36	IbLEA-R	CGGCCGCTGTTTGATTACTG
37	IbCAT-F	TCCACAGGGATGAAGAGGTT
38	IbCAT-R	GGAGCCCAGGATCTGTATCT
39	IbAAO-F	GTCGTTTATGCGGGCTCCT
40	IbAAO-R	CCTTTTCGTCCACCGATTTT
41	IbABA2-F	CTATCGCAAAGTGGATCTGG
42	IbABA2-R	CAAAGCACCCTGCAATAACT

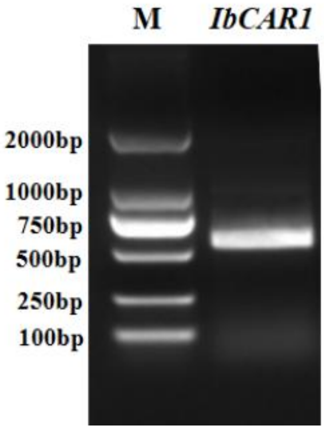

Figure S1. Electrophoresis analysis of *IbCAR1*.

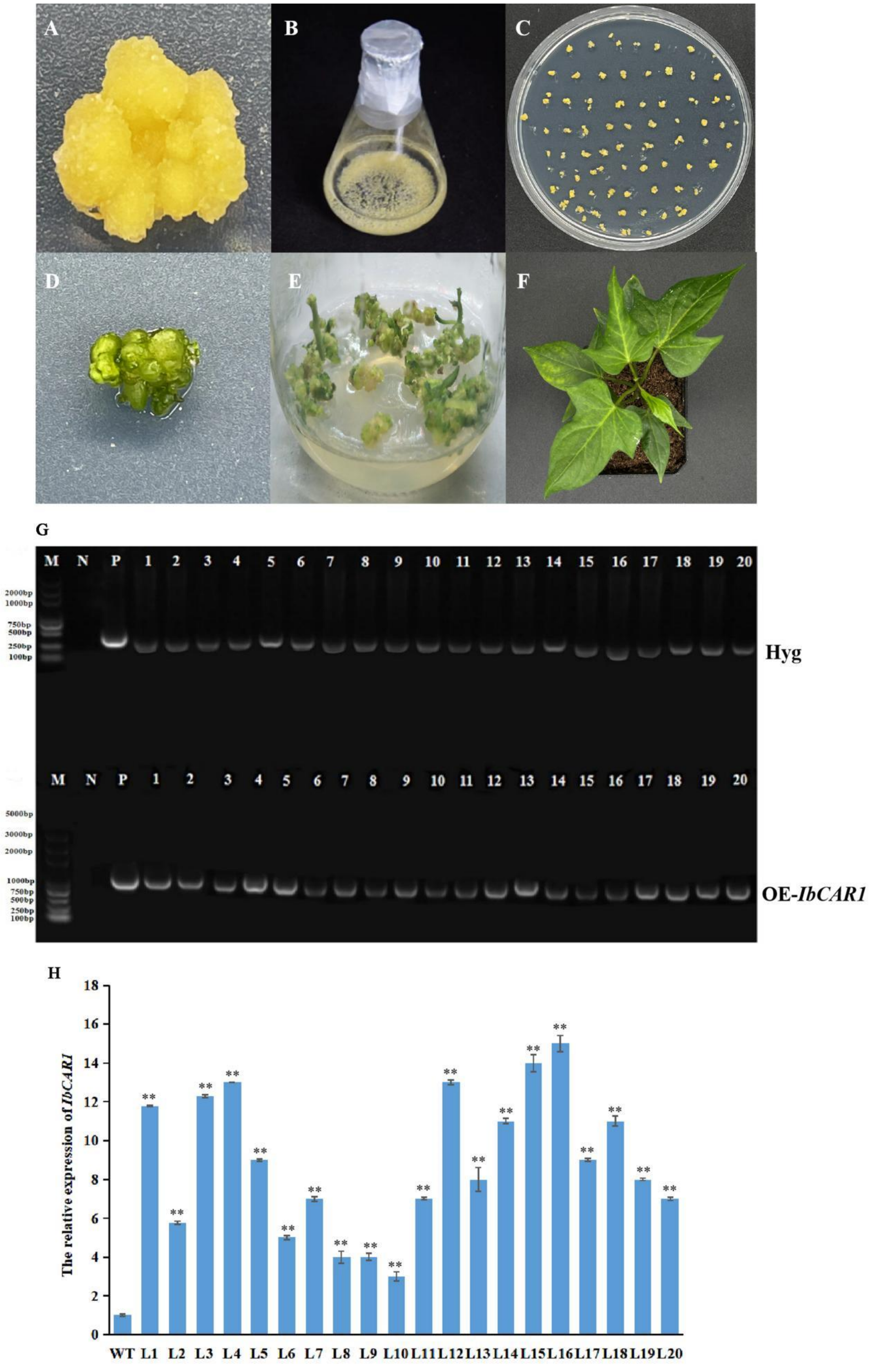

Figure S2. Production of the *IbCAR1*-overexpression sweetpotato plants.

(A) embryonic callus of Xuzishu8. **(B)** embryonic callus in co-culture medium. **(C)** selection of Hyg-resistant embryonic callus. **(D)** ABA inducted 14d. **(E)** Pseudotransgenic seedling. **(F)** Xuzishu8 transgenic lines. **(G)** PCR results of Hyg gene and vector fragment in transgenic plants. P is a positive plasmid as a positive control, N is non-transgenic Xuzishu8 as a negative control. **(H)** Real-time RT-PCR analysis of *IbCAR1* relative expression in overexpressing transgenic sweetpotato leaves. The results are expressed as relative values with respect to the transcript level of the WT, which was set to 1.0. Data are presented as means ± SE (n = 3). ** and * indicate significant differences between the transgenic lines and WT at $p<0.01$ and $p<0.05$ levels, respectively.

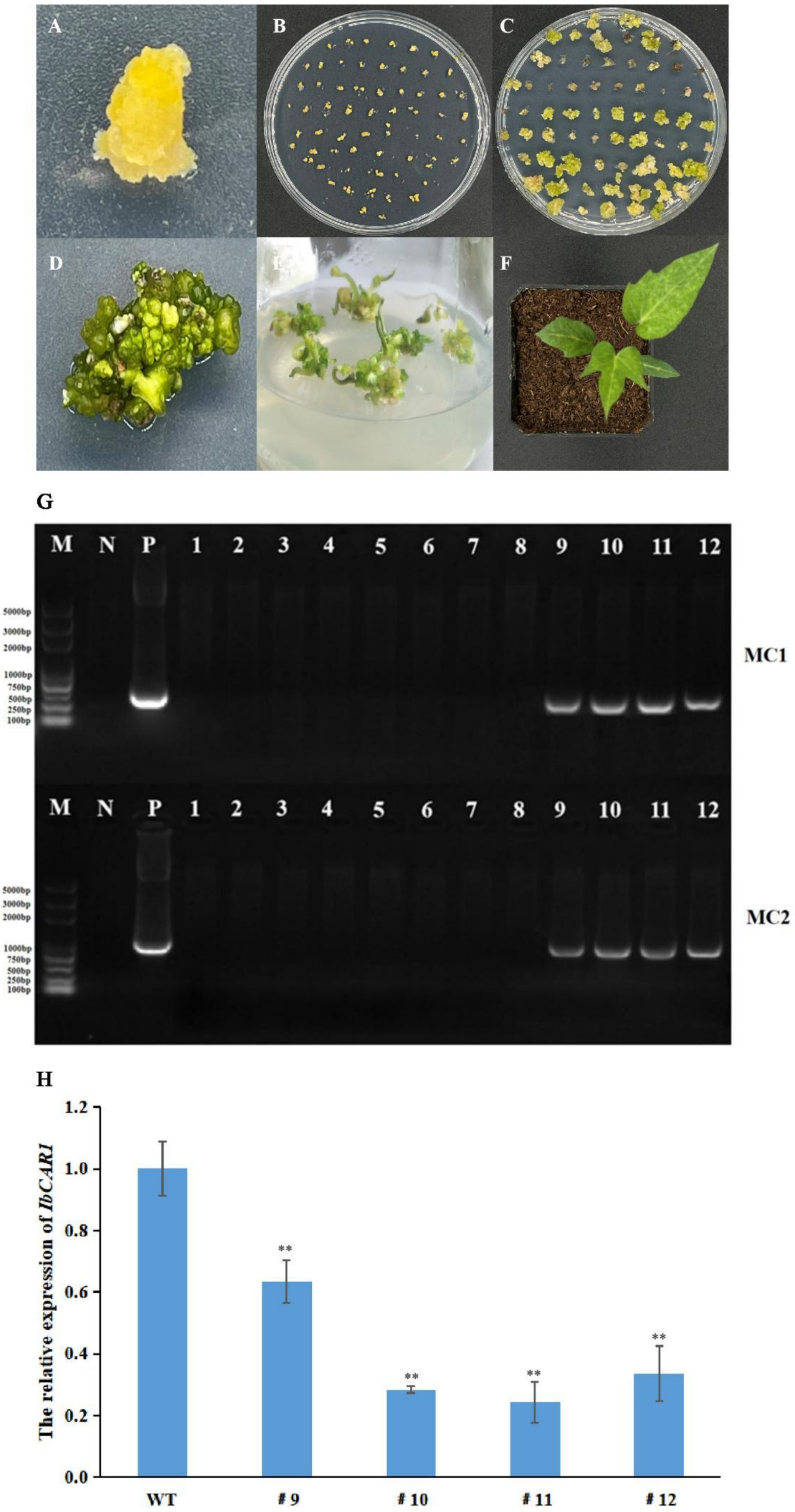

A
B
C
D
E
F
G
M N P 1 2 3 4 5 6 7 8 9 10 11 12
5000bp
3000bp
2000bp
1000bp
750bp
500bp
250bp
100bp
MC1
MC2
H
The relative expression of *IbCAR1*
1.2
1.0
0.8
0.6
0.4
0.2
0.0
**
WT
9
10
11
12

Figure S3. Production of the *IbCAR1*-RNAi sweetpotato plants. **(A)** embryonic callus of Xuzishu8. **(B)** selection of resistant callus. **(C-D)** ABA inducted 14d. **(E)** Pseudotransgenic seedling. **(F)** Xuzishu8 transgenic lines. **(G)** PCR results of MCS1 and MCS2 in putative transgenic plants. P is a positive plasmid as a positive control, N is non-transgenic Xuzishu8 as a negative control. MCS stands for multiple cloning site. **(H)** Real-time RT-PCR analysis of *IbCAR1* relative expression in RNAi transgenic sweetpotato leaves. The results are expressed as relative values with respect to the transcript level of the WT, which was set to 1.0. Data are presented as means ± SE (n = 3). ** and * indicate significant differences between the transgenic lines and WT at $p<0.01$ and $p<0.05$ levels, respectively.
